# Brief Psychotic Episodes and Depressed Mood in a Patient With Borderline Personality Disorder and Antiphospholipid Syndrome

**DOI:** 10.7759/cureus.68382

**Published:** 2024-09-01

**Authors:** William Borchert, James L Megna, Luba Leontieva

**Affiliations:** 1 College of Medicine, State University of New York Upstate Medical University, Syracuse, USA; 2 Psychiatry and Behavioral Sciences, State University of New York Upstate Medical University, Syracuse, USA; 3 Psychiatry, State University of New York Upstate Medical University, Syracuse, USA

**Keywords:** antiphospholipid syndrome, borderline personality disorder, stress-related mental disorders, depressed mood, brief psychotic episode

## Abstract

Psychiatric disorders are reported to be associated with systemic inflammatory conditions and autoimmune diseases. Antiphospholipid syndrome (APS) is a rare condition with poorly understood prevalence and incidence in the general population. Case reports have described co-occurrences of psychiatric conditions and APS. Previous case reports have indicated that patients with APS can have comorbid psychosis, anxiety, depression, and other psychiatric conditions. The association between APS and psychiatric illness, however, remains under-investigated in longitudinal studies.

In this report, we present the case of a woman in her 40s who was voluntarily admitted to the psychiatric inpatient unit for treatment of auditory hallucinations within the context of borderline personality disorder. She reported a rather extensive medical and psychiatric history of several previous illnesses, musculoskeletal injuries, and hospitalizations. Due to the significant social stress and multiple comorbidities, she may be at increased vulnerability to acute exacerbations of both APS and brief psychotic episodes. In this case report, the patient had a history of three hypercoagulability incidents that were shortly followed by psychiatric admissions. This report highlights the importance of considering systemic conditions such as APS in patients presenting with psychiatric illness. Patients with APS and concomitant psychosis may benefit from screening for APS flares in the case of a psychotic break.

## Introduction

Antiphospholipid syndrome (APS) is commonly viewed as an immune system hematological pathology whereby autoantibodies attack phospholipid-binding proteins to form arterial and venous thrombi throughout the entire body. Although commonly viewed within the context of hematology, systemic and neuropsychiatric effects of APS are common. Its relationship with psychiatric conditions, however, is poorly understood.

The incidence and prevalence of APS are not well-described in the literature. The majority of research has focused on placental loss and acute cardiovascular events [1]. In the general population, previous reports have suggested that the annual incidence of APS is approximately 1-2 per 100,000 population, and prevalence is approximately 40-50 per 100,000 population [2-4].

Diagnosis of APS requires that both clinical and laboratory criteria be met. Briefly, there must be one episode of vascular thrombosis or pregnancy morbidity, and the presence of lupus anticoagulant, anticardiolipin, or anti-β2 glycoprotein-I [5]. Neuropsychiatric manifestations of APS exist, and closely related autoimmune conditions such as systemic lupus erythematosus (SLE), have also been associated with brief psychotic episodes and affective disorders such as depression [6-8]. Common neuropsychiatric manifestations of APS range from transient ischemic attacks, strokes, headaches, chorea, seizures, and cognitive dysfunction [6-8]. Few studies to date have investigated APS within the context of personality disorders as a potential neuropsychiatric mechanism. Although the pathophysiology of neuropsychiatric manifestations of APS is not well understood, most literature points towards thrombus formation being the primary mechanism by which APS affects the nervous system [8]. Additionally, there is some evidence that anticardiolipin may bind to the neurons themselves [8].

Diagnostic and Statistical Manual of Mental Disorders, Fifth Edition (DSM-V) criteria for borderline personality disorder (BPD) state that the disorder is characterized by pervasive instability in one's affects, self-image/worth, and interpersonal relationships [9]. Additionally, impulsivity, fear of abandonment, suicidal behavior and self-harm, sense of emptiness, depressive symptoms, and paranoia are common presentations of BPD. Common differential diagnoses for BPD are depressive or bipolar disorders, other personality disorders (e.g., histrionic, antisocial, or paranoid), substance use disorders, identity problems (e.g., adolescence), and personality changes due to a medical condition [9]. BPD has been associated with systemic inflammation and somatic conditions [10,11]. Therefore, we present a case of a patient with BPD experiencing auditory hallucinations and depressed mood.

## Case presentation

The patient is a self-identified female in her 40s who presents to the Emergency Department (ED) with complaints of psychiatric distress and a reported history of BPD, anxiety, depression, post-traumatic stress disorder (PTSD), and cocaine use disorder in early remission. She reported hearing "voices" and "noise" which would constantly tell her that she is not good enough, that she will not succeed in life, and command hallucinations telling her to kill herself. She complained of a chronic sense of feeling "not good enough," even in the absence of the noise, and a fear of being abandoned by her boyfriend despite a desire to end the relationship due to his substance use and physical abuse towards her. Upon chart review, her history was more consistent with BPD and PTSD. She was amenable to voluntary psychiatric admission for command hallucinations telling her to kill herself and a depressed mood in the context of worsening stressors at home.

Family history

The patient did not report any formally diagnosed mental illnesses in first-degree relatives although she did report a history of depression on both sides of the family. Notably, she does not report a family history of autoimmune conditions.

Social history

She met developmental milestones on time with reasonable accommodations for math and reading and was able to briefly attend college. Throughout childhood, she reported bullying but no significant trauma until she reached adulthood and began living with significant others. She has worked in several healthcare settings and has been a certified nursing assistant and patient care technician. She reported significant emotional abuse and trauma from several significant others exacerbated by concomitant use of crack cocaine for the past decade. Out of all psychosocial stressors, she reports a significant other being the main source of stress at her home, and she remains at risk for intimate partner violence. She is socially integrated through work, extended family, and friends, and maintains stable employment and adequate transport to her outpatient appointments, thus limiting financial strain. The patient recently completed a 28-day in-person rehabilitation for crack-cocaine use and had been over 90 days sober at the time of the admission. She has been attending Narcotics Anonymous and was future-oriented about maintaining sobriety. The patient reported using coping mechanisms of meditation and self-talk to limit the "noise" from the defeatist internal monologue.

Past medical and psychiatric history

Throughout the course of her life, she reports over 20 visits to the ED for a variety of medical and psychiatric issues ranging from gastroenteritis, biliary colic, musculoskeletal pain, headache, depression, psychosis, and injuries. Since the diagnosis of APS, she continued to present to the ED with both medical and psychiatric complaints. In particular, there were three occurrences of medical conditions with increased hypercoagulable risk (e.g., pulmonary embolism, surgery) which were shortly followed by unspecified psychosis. Upon conversation with the patient, she provided a detailed sequence of events regarding her previous psychiatric hospitalizations and medical hospitalizations which preceded the psychiatric admissions. She has previously tried sertraline which induced hallucinations, and citalopram and escitalopram which did not work for her psychiatric symptoms.

In 2021, she presented to the ED with multiple pulmonary embolisms and was subsequently diagnosed with APS and anemia approximately two weeks later. There was no prior diagnosis of any coagulopathies or autoimmune disorders. Her history is unremarkable for SLE or other hematological conditions.

Two weeks after her admission for pulmonary embolisms and diagnosis of APS, she presented with command hallucinations to the ED. She reported that the auditory hallucinations were exacerbated by her stress following the previous hospitalization and diagnosis. Approximately one year later, she again reported to the ED with acute cholecystitis which was followed again by command hallucinations and a profound sense of worthlessness shortly after cholecystectomy, ultimately resulting in psychiatric hospitalization. Most recently, the patient sustained an injury from a fall in 2023, which resulted in a closed fracture of the foot and subsequent surgery. Within two weeks of the surgery to remove the plate, she presented to the ED with command auditory hallucinations. These auditory hallucinations are negative in nature, and the patient reports a feeling of worthlessness and hopelessness from the voices. The patient describes these hallucinations as "noise" and people telling her that she is "not good enough" and "will not be successful" in life, but she is unable to identify any specific person whom she believes to be the source of her auditory hallucinations.

**Figure 1:**
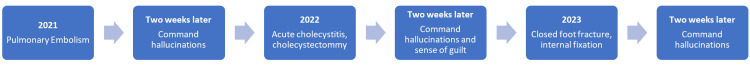
Timeline of APS-related events and psychiatric admissions APS: antiphospholipid syndrome

As described above, in a sequence of three admissions for psychiatric illness, she presented to the ED with an APS-related complication within the two weeks prior to each psychiatric admission. The sequence of APS-related thrombophilic event events along with the psychiatric symptoms are depicted in Figure 1.

The patient was voluntarily admitted from the ED for a self-described mental health crisis for a period of 10 days. She reported a depressed mood accompanied by the command hallucinations self-described as "voices" and "noise" without being able to elaborate further. She reports that stress can increase her perception of the voices that exacerbate her mood symptoms which are present at baseline. She did not report voices saying anything specific but provided vague references to the voices continuing to vex her with a profoundly negative self-image and a sense of abandonment from those around her. She was admitted for stabilization and treatment of command auditory hallucinations and depressed mood.

Interventions in the unit

**Figure 2:**
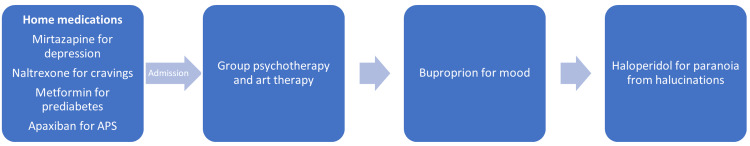
Interventions in the unit APS: antiphospholipid syndrome

Throughout the course of hospitalization, the patient continued with her previous medications such as 380mg/vial naltrexone extended-release injection for cravings, 7.5mg mirtazapine for depression, and 500mg metformin for prediabetes. She reports good control of cravings with the long-acting naltrexone injectable, which was due for administration during the hospital course and provided to the patient. She follows with hematology after having an unprovoked pulmonary embolism and positive lupus anticoagulant. She was continued on 2.5mg apixaban twice daily throughout the hospitalization for APS in order to maintain anticoagulation. In addition to the preexisting medications, she was started on 150mg bupropion daily for mood and 1mg haloperidol as needed for paranoia associated with the command hallucinations. The sequence of these interventions in the unit is depicted in Figure 2. She reported rapid improvement in her symptoms of depressed mood after the addition of bupropion to the regimen. Likewise, the auditory hallucinations were diminished in frequency and intensity and had nearly subsided after three days of as-needed haloperidol. After stabilization, she reported that over the course of the hospitalization, she had less need to take haloperidol regularly to suppress the "noise."

Prior to this most recent hospitalization, her hypertriglyceridemia was not treated. Medicine was consulted and she began treatment with 48mg of fenofibrate daily due to triglycerides being 584mg/dL (normal <150mg/dL). The patient participated in group psychotherapy sessions extensively throughout hospitalization and implemented several coping skills to minimize the "noise." These sessions included stress management techniques, implementation of coping skills, emotional regulation, distress tolerance, mindfulness, and interpersonal effectiveness.

At discharge, her diagnoses were unspecified psychotic disorder, PTSD, BPD, and cocaine use disorder in early remission. We considered other diagnoses such as major depressive disorder, and other personality disorders, but her current and past clinical history were most consistent with BPD due to her substance use being in early remission, the efforts to avoid abandonment, unstable self-image, chronic feelings of emptiness, unstable interpersonal relationships, and history of self-injurious substance use. Long-term management for this patient after discharge included continuing with her standing and as-needed psychiatric medications targeting depression and auditory hallucinations, maintenance of cravings with naltrexone, continued participation in Narcotics Anonymous, and regular outpatient cognitive behavioral therapy.

## Discussion

This case represents a unique presentation of psychotic and mood symptoms in the context of BPD and comorbid APS. Moreover, the patient in this case had three occurrences of psychiatric hospitalizations which were preceded by hypercoagulability exacerbations within the previous month each time. Inflammation and stress have been posited to play a role in BPD [10], and the exact mechanisms by which systemic inflammation affects the brain remain under active investigation. Likewise, there have been several reports of patients with APS presenting with depression, anxiety, and psychosis [6].

That a biological mechanism involved in APS could affect psychiatric health is plausible. SLE, a similarly situated autoimmune condition with significant hematological sequelae, also has been associated with psychosis [12]. Although APS and SLE commonly are associated with strokes, there remains the possibility that inflammation on the cellular level of the neuron is ongoing in these conditions which would not be detectable in routine imaging. Antiphospholipid antibodies bound to neurons and glial cells have been detected in both animals and in vitro models [13,14], which points toward a potential mechanism for neuropsychiatric manifestations of APS.

Proper management of comorbidities arising from APS may be a critical intervention in preventing the negative downstream effects of this disease. Moreover, a systematic review of psychosis cases and APS has suggested that sudden, late-onset psychosis in middle-aged female patients may raise suspicion for APS [15]. Therefore, it is critical that providers recognize that inflammatory conditions can go in parallel with psychiatric conditions. If there are no other systemic inflammatory conditions, APS is usually managed by anticoagulation only and standard follow-up tests are usually limited to monitoring anticoagulation [16].

There remains the possibility that the stressful events in the patient's personal life or health are parts of the sequelae leading up to an acute psychiatric presentation and that APS is merely coincidental. Moreover, the possibility that the temporality of the association is reversed, meaning that psychosis precedes APS, is likewise plausible. Likewise, it has been proposed that adverse childhood experiences could contribute to autoimmune conditions in adulthood, but meta-analyses have not shown a consistent relationship [17]. Adverse childhood experiences, however, have been strongly linked to the development of BPD, which may indicate that long-term exposure to stress in childhood increases the risk for BPD [18].

This case has several limitations which are inherent with the nature of case reports. Notably, the patient's presentation may not be generalizable to other cases, and there may be alternative explanations for the concomitant presentation of BPD and APS. This case report does not claim that APS plays a causal role in brief psychotic episodes and exacerbations of underlying psychiatric conditions. The potential for reverse causation is also possible in that a psychiatric illness such as BPD could lead to more stress and injurious behavior which leads to a chronic inflammatory state thereby increasing the risk for conditions such as APS. Additionally, the depressed mood and psychotic symptoms in this case are within the context of BPD, which may limit the generalizability of an association of APS with psychosis or affective disorders. Nonetheless, there is an increasing body of evidence on comorbid autoimmune and psychiatric conditions. In those with APS, patient education in recognizing the symptoms of psychiatric conditions may represent an important early intervention strategy.

Studies establishing the prevalence of APS and concomitant psychosis are necessary to determine if psychosis in patients with APS exceeds population norms. Likewise, in order to better understand the temporality of APS and psychotic episodes, longitudinal studies, both prospective and retrospective, can shed light on the potential etiology of rare hematological conditions and brief psychotic breaks.

## Conclusions

APS is an underreported disorder in the population with its prevalence and incidence poorly understood. APS may be an important predisposing condition that plays a role in the development of psychiatric disorders such as BPD, psychosis, and affective disorders. Moreover, it is important to screen for psychiatric symptoms in the context of coagulopathies caused by APS and SLE in order to provide proper interdisciplinary care. Longitudinal observational studies may provide further insight into the temporality and association between APS and psychiatric conditions.
